# Registro Nacional do Controle da Hipertensão Arterial Avaliado pela Medida de Consultório e Residencial no Brasil: Registro LHAR

**DOI:** 10.36660/abc.20220863

**Published:** 2023-07-21

**Authors:** Roberto Dischinger Miranda, Andréa Araujo Brandão, Weimar Kunz Sebba Barroso, Marco Antonio Mota-Gomes, Eduardo Costa Duarte Barbosa, Lucio Paulo Ribeiro, Claudinelli Alvarenga Aguilar, Fabio Serra Silveira, Cristiano de Melo Rangel Gomes, Abraham Epelman, Annelise Machado Gomes de Paiva, Audes Diógenes Magalhães Feitosa

**Affiliations:** 1 Escola Paulista de Medicina Universidade Federal de São Paulo São Paulo SP Brasil Serviço de Cardiologia, Disciplina de Geriatria e Gerontologia, Escola Paulista de Medicina, Universidade Federal de São Paulo, São Paulo, SP – Brasil; 2 Universidade do Estado do Rio de Janeiro Rio de Janeiro RJ Brasil Universidade do Estado do Rio de Janeiro, Rio de Janeiro, RJ – Brasil; 3 Liga de Hipertensão Arterial Hospital das Clínicas Universidade Federal de Goiás Goiânia GO Brasil Liga de Hipertensão Arterial - Hospital das Clínicas - Universidade Federal de Goiás, Goiânia, GO – Brasil; 4 Centro Universitário CESMAC Hospital do Coração Maceió AL Brasil Centro Universitário CESMAC, Hospital do Coração, Maceió, AL – Brasil; 5 Liga de Combate à Hipertensão Arterial Porto Alegre RS Brasil Liga de Combate à Hipertensão Arterial, Porto Alegre, RS – Brasil; 6 Beliva Recife PE Brasil Beliva, Recife, PE – Brasil; 7 Hospital Clínica do Esporte Goiânia GO Brasil Hospital Clínica do Esporte, Goiânia, GO – Brasil; 8 Centro de Pesquisa Clínica do Coração Aracaju SE Brasil Centro de Pesquisa Clínica do Coração, Aracaju, SE – Brasil; 9 Clínica Dicor Saquarema RJ Brasil Clínica Dicor, Saquarema, RJ – Brasil; 10 Servier do Brasil Rio de Janeiro RJ Brasil Servier do Brasil, Rio de Janeiro, RJ – Brasil; 11 Unidade de Hipertensão e Cardiologia Preventiva PROCAPE Universidade de Pernambuco Recife PE Brasil Unidade de Hipertensão e Cardiologia Preventiva do PROCAPE, Universidade de Pernambuco, Recife, PE – Brasil

**Keywords:** Hipertensão, Pressão Arterial, Monitorização Residencial da Pressão Arterial, Controle Pressórico

## Abstract

**Fundamento:**

Sabe-se que em torno de 30% dos pacientes apresentam valores de pressão arterial (PA) mais elevados quando examinados no consultório do que em suas residências. No mundo, admite-se que apenas 35% dos hipertensos já tratados tenham alcançado meta pressórica.

**Objetivo:**

Fornecer dados epidemiológicos sobre o controle da PA nos consultórios, em uma amostra de cardiologistas brasileiros, avaliado pela medida de consultório e monitorização residencial da pressão arterial (MRPA).

**Métodos:**

Análise transversal. Observou-se pacientes com diagnóstico de hipertensão arterial, em tratamento anti-hipertensivo, podendo ou não estar com a PA controlada. A PA foi verificada no consultório por profissional médico, e no domicílio através da MRPA. A associação entre variáveis categóricas se deu por meio do teste do qui-quadrado (p < 0,05).

**Resultados:**

Foram incluídos 2.540 pacientes, com idade média 59,7 ± 15,2 anos. A maioria dos pacientes eram mulheres (62%; n = 1.575). O estudo mostrou uma prevalência de 15% (n = 382) de hipertensão do avental branco não controlada, e 10% (n = 253) de hipertensão mascarada não controlada. A taxa de controle da PA no consultório foi 56,3%, e no domicílio, de 61%; 46,4% dos pacientes tiveram PA controlada no consultório e fora dele. Observou-se maior controle no sexo feminino e na faixa etária 49-61 anos. Observando o controle domiciliar com o novo ponto de corte das Diretrizes Brasileiras de Hipertensão Arterial de 2020, a taxa de controle foi de 42,4%.

**Conclusão:**

O controle pressórico nos consultórios em uma amostra de cardiologistas brasileiros foi de 56,3%; 61% quando a PA foi obtida no domicílio, e 46,4% quando o controle foi observado tanto no consultório como no domicílio.

## Introdução

A hipertensão arterial (HA) é uma condição crônica definida por valores persistentemente elevados de pressão arterial (PA) que, se não devidamente controlados, gera repercussões sistêmicas causadas por lesões estruturais e/ou funcionais a órgãos-alvo. A HA é o principal fator de risco modificável para os eventos cardiovasculares e cerebrovasculares. É considerada um importante problema de saúde pública por apresentar prevalência alta e crescente, baixos índices de controle e morbidade/mortalidade elevadas.^1 - 4^ A frequência autorreferida de diagnóstico médico de HA na população adulta das capitais brasileiras e Distrito Federal é de 25,2%, sendo maior entre mulheres (26,2%) do que entre homens (24,1%). Em ambos os sexos, essa frequência aumentou com a idade e diminuiu com o nível de escolaridade.^5^

A mensuração da PA é, naturalmente, imprescindível para o diagnóstico. No entanto, apesar de ser um procedimento simples, podem ocorrer erros durante sua medição, sejam relacionados ao aparelho, à técnica, à influência do ambiente, ao próprio paciente ou, ainda, ao observador.^6 , 7^ O diagnóstico de HA é dado, segundo os novos critérios da diretriz americana, quando o indivíduo apresenta valor de PA sistólica (PAS) ≥ 130 mmHg e/ou PA diastólica (PAD) ≥ 80 mmHg, tanto em medida no consultório quanto domiciliar e ambulatorial.^8^ A última Diretriz Brasileira, a Europeia de 2018, como também a Diretriz da Sociedade Internacional de HA 2020, mantêm os critérios anteriores, considerando como hipertenso o indivíduo que apresenta PAS ≥ 140 e/ou PAD ≥ 90 mmHg para medidas em consultório. A Diretriz Europeia e Brasileira trás, no entanto, mudanças nas recomendações sobre quando se considerar o início do tratamento medicamentoso de acordo com o risco cardiovascular.^1 , 9 , 10 , 11^

Sabe-se que porcentagem significativa – em torno de 30% – dos pacientes apresenta valores de PA mais elevados quando examinados no ambiente de consultório do que em suas residências.^12 - 14^ A hipertensão do avental branco (HAB) ocorre quando há elevação pressórica persistente no ambiente assistencial e valor normal fora dele, levando à superestimação dos níveis de PA do paciente e consequente erro no diagnóstico da HA. O oposto da HAB ocorre quando o paciente apresenta níveis pressóricos dentro dos limites de normalidade em medida realizada no consultório, porém PA elevada fora dele, o que caracteriza a hipertensão mascarada (HM). Para que seja possível diferenciar a HAB da hipertensão sustentada ou, ainda, para que se identifique a presença da HM, é necessário que se faça a medição da PA do paciente fora do ambiente médico. Os métodos atualmente utilizados são a monitorização ambulatorial da pressão arterial (MAPA) e a monitorização residencial da pressão arterial (MRPA).^13 - 17^

A MRPA é o registro da PA realizado durante a vigília pelo paciente ou outra pessoa treinada, através de aparelho automático, por vários dias, fora do ambiente de consultório, com número de medidas e horários previamente determinados. A MRPA demonstrou ser o método diagnóstico da HA que melhor atua na eliminação dos efeitos anteriormente citados,^18^ com as vantagens adicionais de apresentar boa reprodutibilidade, boa capacidade prognóstica, avaliação do efeito do tratamento em diferentes períodos do dia, custo relativamente baixo e boa aceitação pelo paciente. Uma revisão sistemática concluiu que tanto a baixa sensibilidade da medida de consultório para detectar o controle ótimo da PA quanto a associação da MRPA com mortalidade cardiovascular apoiam o uso rotineiro desta última na prática clínica.^19^ Estudos demonstraram que a utilização da MRPA no seguimento do paciente hipertenso está associada à melhor adesão ao tratamento medicamentoso, com consequente melhora no controle da PA e redução nos desfechos cardiovasculares quando comparada com a PA medida no consultório.^7 , 20^

No mundo, admite-se que menos de 15% da população total de hipertensos tenham alcançado a meta pressórica recomendada, e essa taxa é de apenas 35% entre os hipertensos já tratados.^1^ O fato ganha magnitude quando levamos em consideração que as metas pressóricas recomendadas pelas diretrizes mais recentes^1 , 2 , 8 - 11^ tornaram-se mais baixas, o que tende a aumentar o percentual de indivíduos hipertensos não controlados e, por consequência, o risco associado de morbidade e mortalidade de doenças cardiovasculares. O primeiro registro brasileiro de hipertensão,^21^ usando um ponto de corte mais baixo para controle da PA (< 130 × 80 mmHg), encontrou 24,3% da população geral controlados no início da observação, e 24,7% em 1 ano.

Assim, este estudo tem como objetivo fornecer dados epidemiológicos sobre o controle da HA nos consultórios em uma amostra de cardiologistas brasileiros, avaliado pela medida de consultório e residencial (MRPA).

## Métodos

Trata-se de uma análise transversal, realizada em 231 centros particulares de atenção especializada em cardiologia, localizados em 23 estados brasileiros e mais o Distrito Federal, englobando todas as cinco regiões do Brasil, entre junho e dezembro de 2019. A amostra foi obtida por conveniência e constituída por pacientes com diagnóstico médico de HA, atendidos ambulatorialmente, com idade ≥ 18 anos, em tratamento anti-hipertensivo, podendo ou não estar com a PA controlada. Solicitou-se aos médicos investigadores que convidassem para participar da pesquisa sempre o segundo paciente do dia, para evitar um viés de seleção.

Preliminarmente, os participantes foram informados sobre os objetivos e procedimentos da pesquisa e, a seguir, convidados a participar voluntariamente do estudo. Procedeu-se a coleta de dados após a assinatura no Termo de Consentimento Livre e Esclarecido. O referido estudo foi aprovado pelo Comitê de Ética em Pesquisa Humana do Hospital das Clínicas da Universidade Federal de Goiás com o CAEE: 08208619.8.0000.5078.

Foram coletados dados demográficos, clínicos e antropométricos. As variáveis data de nascimento, idade, sexo e uso ou não de medicação anti-hipertensiva foram coletadas através de questionário durante o atendimento. O peso e altura foram obtidos usando balanças antropométricas devidamente calibradas e validadas, e o índice de massa corpórea (IMC) de adultos classificado segundo a Organização Mundial da Saúde.^22^

A medida da PA de consultório foi realizada por profissional médico, segundo as recomendações das VII Diretrizes Brasileiras de Hipertensão Arterial (VII DBHA),^1^ utilizando-se manguito apropriado em conformidade com as dimensões do braço do indivíduo. Não participaram do estudo pacientes com arritmia e circunferência do braço > 42 cm e < 22 cm, devido às limitações do medidor de PA.

A MRPA foi obtida segundo as orientações da IV Diretriz de Monitorização Residencial da Pressão Arterial e da Diretriz Europeia de Hipertensão Arterial.^7 , 9^ Dessa forma, foram adquiridas duas medidas no primeiro dia, ainda no ambiente do ambulatório (essas medidas não foram utilizadas para análise da média residencial), e medidas domiciliares de 4 dias consecutivos, com três medidas pela manhã e três medidas no horário noturno, totalizando 24 medidas. Os pacientes foram instruídos a realizar as medidas conforme protocolo e anotar num diário de PA para aumentar a adesão à metologia da MRPA. As medidas, também, foram registradas e armazenadas na memória do equipamento e posteriormente inseridas na plataforma TeleMRPA^®^, uma ferramenta de laudo a distância por telemedicina. Tanto a medida da PA de consultório como a MRPA foram obtidas através do equipamento eletrônico HEM 7320 (Omron Healthcare Co. Ltd., Kyoto Japan).

Considerou-se HA não controlada, com base na medida do consultório, daqueles participantes que tinham PAS ≥ 140 mmHg e/ou PAD ≥ 90 mmHg, e com base na MRPA ≥ 135 mmHg para PAS e/ou ≥ 85 mmHg para PAD. Adicionalmente, analisamos a taxa de controle domiciliar com base nos novos valores de corte para MRPA, recomendados pela DBHA 2020^11^ . Utilizou-se os termos HM não controlada (HMNC) para aqueles participantes da pesquisa que tiveram PA controlada no consultório, mas PA domiciliar ou ambulatorial elevada; HAB não controlada (HABN) para aqueles que tiveram a PA elevada no consultório, mas PA controlada residencial ou ambulatorial, e hipertensão sustentada (HS) para aqueles que tiveram a PA não controlada no consultório e ambulatorial. Embora os termos HAB e HM fossem, originalmente, definidos para pessoas que não estavam sendo tratadas para HA, nos últimos tempos, também, são usadas para descrever discrepâncias entre a PA no consultório e fora do consultório em pacientes tratados para HA.^9 , 23^

O banco de dados foi estruturado em Excel® (Microsoft) com os dados da MRPA importados da plataforma de registro, assim como os demais dados coletados pelos investigadores. As variáveis contínuas estão apresentadas como média e desvio padrão, enquanto as categóricas como frequências relativas e absolutas. A associação entre variáveis categóricas se deu por meio do teste do qui-quadrado. Adotou-se um valor de significância de p < 0,05. Foi utilizado o programa estatístico SPSS v. 21.0 (IBM Inc., Chicago, IL, EUA).

## Resultados

A amostra estudada foi de 2.540 pacientes, sendo 1,9% (n = 49) dos participantes da pesquisa da região Norte, 18% (n = 458) da região Nordeste, 58,2% (n = 1479) da região Sudeste, 13,5% (n = 342) da região Sul, e 8,3% (n = 211) da região Centro-Oeste. Desses, 1.575 (62%) eram do sexo feminino, e 965 (38%), do sexo masculino. A idade média foi de 59,7 ± 15,2 anos, e o IMC médio, de 28,6 ± 5,1 kg/m^2^ ( Tabela 1 ).

**Tabela 1:** – Características descritivas da amostra (n = 2.540)

Variável	n	%
**IMC**		
Desnutrição	23	0,9
Eutrofia	590	23,2
Sobrepeso	1.070	42,1
Obesidade	857	33,7
**Sexo**		
Feminino	1.575	62,0
Masculino	965	38,0
	**Média**	**DP**
Idade (anos)	59,7	15,2
IMC (kg/m^2^)	28,6	5,1

Fonte: dados da pesquisa. IMC: índice de massa corpórea; DP: desvio-padrão.

Os valores médios da PA de consultório foram de 133,3 ± 20,4 mmHg e 82,3 ± 13,2 mmHg, e pela MRPA as médias foram de 125,9 ± 16,1 mmHg, e 78,6 ± 9,3 mmHg para PAS e PAD, respectivamente. Os participantes da pesquisa tiveram 14 ou mais medidas válidas na MRPA, tendo a maioria (94%) dos participantes um total de 24 medidas válidas. O estudo mostrou uma prevalência de 15% (n = 382) de HABNC e 10% (n = 253) de HMNC ( Tabela 2 ).

**Tabela 2:** – Prevalência dos diferentes fenótipos de hipertensão (n = 2.540)

Fenótipos	n	%
HABNC	382	15
HC	1.168	46
HMNC	253	10
HS	737	29

Fonte: dados da pesquisa. HABNC: hipertenso do avental branco não controlado; HC: hipertenso controlado; HMNC: hipertenso mascarado não controlado; HS: hipertenso sustentado.

A prevalência de HABNC no sexo feminino foi de 16% (n = 252) e HMNC de 8,4% (n = 132), enquanto no sexo masculino essa prevalência foi de 13,5% (n = 130) para HABNC e 12,5% (n = 121) para HMNC. A prevalência de HMNC no sexo masculino foi significativamente maior que no sexo feminino, enquanto o sexo feminino apresentou o maior número de pacientes controlados. Em relação ao IMC não houve diferença estatística entre os fenótipos de hipertensão, e em relação à faixa etária, os mais idosos (quartil 4 = 70-98 anos) apresentaram a maior prevalência de HMNC e o menor controle pressórico ( Tabela 3 ).

**Tabela 3:** – Fenótipos *versus* variáveis

Fenótipo	Sexo								p-valor*

	Feminino (n = 1.575)				Masculino (n = 965)				

	n		%		n		%		
Fenótipo									< 0,01
HABNC	252		16,0		130		13,5		
HC	754		47,9		414		42,9^†^		
HMNC	132		8,4		121		12,5^†^		
HS	437		27,7		300		31,1		
**Fenótipo**	**Índice de massa corpórea**								**p-valor**
	**Desnutrição (n 23)**		**Eutrofia (n 590)**		**Sobrepeso (n 1.070)**		**Obesidade (n 857)**		
	**n**	**%**	**n**	**%**	**n**	**%**	**n**	**%**	
**Fenótipo**									0,24
HABNC	7	30,4	84	14,2	168	15,7	123	14,4	
HC	8	34,8	269	45,6	511	47,8	380	44,3	
HMNC	1	4,3	56	9,5	108	10,1	88	10,3	
HS	7	30,4	181	30,7	283	26,4	266	31,0	
**Fenótipo**	**Idade**								**p-valor**
	**Q1 (n 650) (18-49 anos)**		**Q2 (n 673) (49-61 anos)**		**Q3 (n 587) (61-70 anos)**		**Q4 (n 630) (70-98 anos)**		
	**n**	**%**	**n**	**%**	**n**	**%**	**n**	**%**	
**Fenótipo**									< 0,01
HABNC	97	14,9	115	17,1	88	15,0	82	13,0	
HC	319	49,1	325	48,3	284	48,4	240	38,1^‡^	
HMNC	62	9,5	59	8,8	46	7,8	86	13,7^‡^	
HS	172	26,5	174	25,9	169	28,8	222	35,2^‡^	

*p-valor para o teste do qui-quadrado. ^†^Essa prevalência difere significativamente da prevalência dos indivíduos do sexo feminino. ^‡^Essa prevalência difere significativamente da prevalência das demais colunas. HABNC: hipertenso do avental branco não controlado; HC: hipertenso controlado; HMNC: hipertenso mascarado não controlado; HS: hipertenso sustentado.

A taxa de controle da PA dos participantes da pesquisa no consultório foi 56,3% (n = 1.431). Já no domicílio, observamos um controle de 61% (n = 1.550), enquanto 46,4% (n = 1.178) dos participantes da pesquisa tiveram PA controlada no consultório e fora dele ( Figura central ).

**Figura Central:**
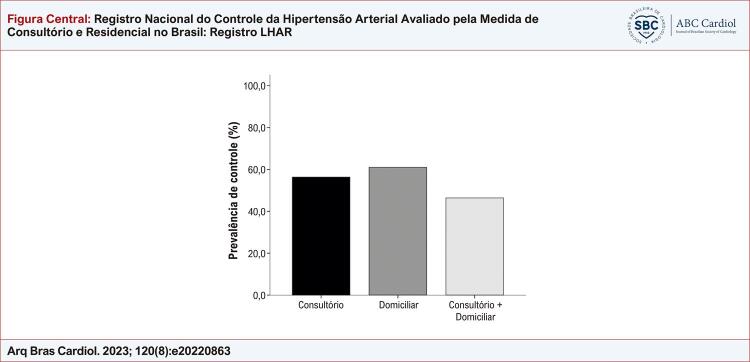
: Registro Nacional do Controle da Hipertensão Arterial Avaliado pela Medida de Consultório e Residencial no Brasil: Registro LHAR

Prevalência de controle geral.

**Figura 1:**
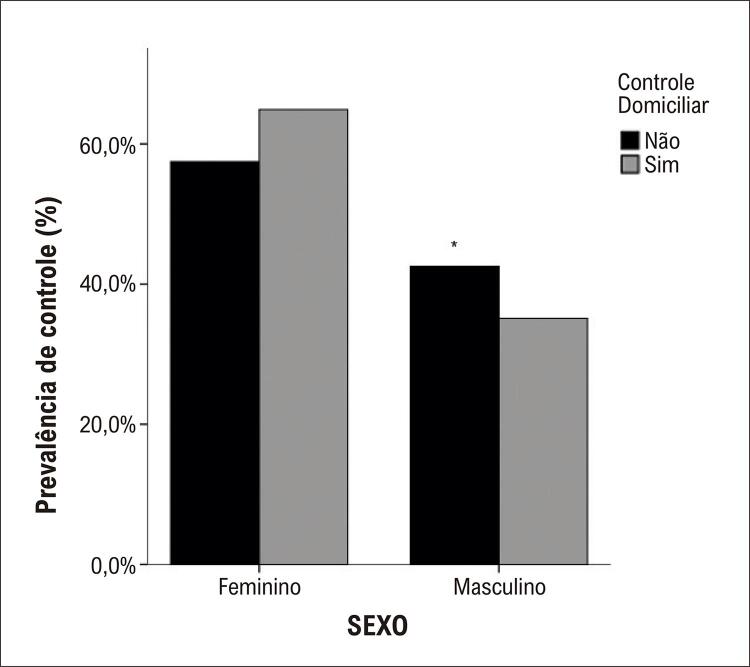
– Prevalência de controle pressórico no domicílio estratificado por sexo (p < 0,05).

**Figura 2:**
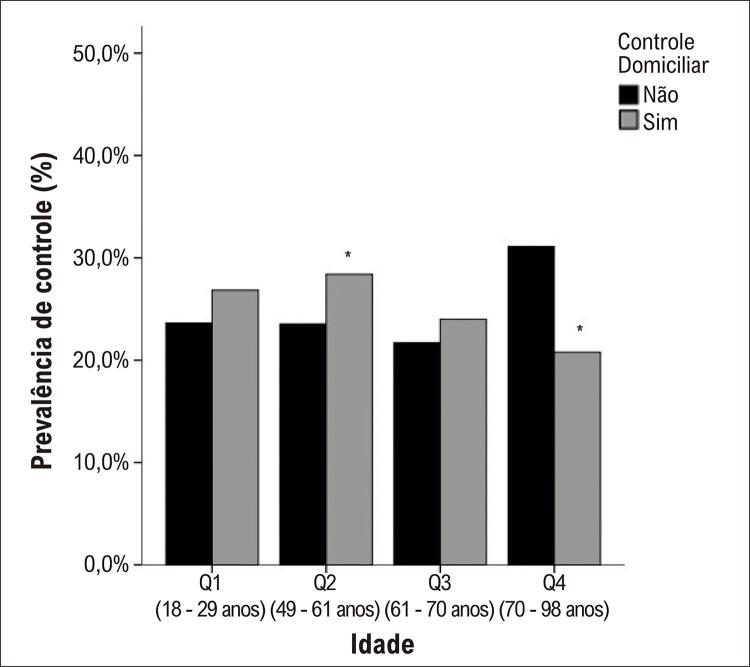
– Prevalência de controle pressórico no domicílio estratificado por idade (p < 0,05).*

Observando o controle domiciliar estratificado pelo sexo ( Figura 1 ) e idade ( Figura 2 ), encontrou-se maior controle no sexo feminino e no quartil 2 (faixa etária 49-61 anos), respectivamente.

A Diretriz Brasileira de Hipertensão 2020^11^ propôs como valores de normalidade para MRPA 130 mmHg para PAS e 80 mmHg para PAD. Observando esse novo ponte de corte, a prevalência de paciente HABNC foi de 7,6% (n = 194), HC 34,9% (n = 886), HMNC 21,8% (n = 553) e HS 35,7% (n = 907).

**Figura 3:**
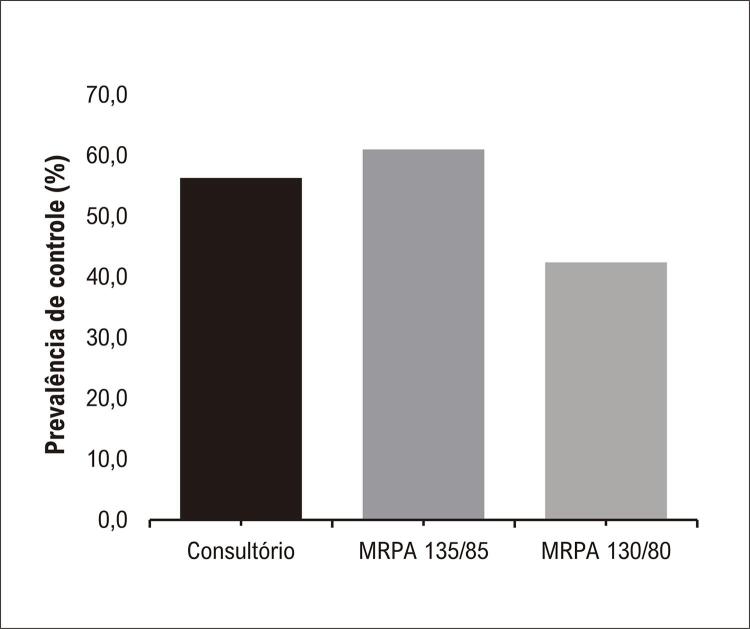
– Prevalência de controle com os novos pontos de corte para monitorização residencial da pressão arterial.

A Figura 3 expõe a prevalência de controle pressórico no consultório e no domicílio, observando o pontos de corte atual e o proposto anteriormente.

## Discussão

O diagnóstico e tratamento da HA têm sido baseado principalmente na medição da PA no consultório. No entanto, a PA pode diferir consideravelmente quando medida no consultório e quando medida fora do ambiente do consultório,^24^ sendo que uma PA mais alta fora do consultório está associada a risco cardiovascular aumentado, independente da PA do consultório.

O presente estudo evidenciou que os indivíduos apresentaram PA mais elevada no consultório que as obtidas em domicílio. Sabe-se que as medidas da MRPA geralmente são mais baixas do que as realizadas no consultório e mais próximas da pressão média registrada durante a MAPA de 24 horas.^25^

A totalidade da amostra estudada obteve o número de medidas válidas na realização da MRPA. Uma MRPA com boa qualidade depende fundamentalmente das orientações fornecidas ao paciente e da utilização de um diário de medidas, que eliminam, em quase 100%, a necessidade de repetição de exames por número insuficiente de medidas.^13 , 26^

A MRPA fornece informações importantes sobre os níveis da PA fora do ambiente do consultório, em diferentes momentos do dia. Uma das grandes vantagens da MRPA é a identificação e acompanhamento dos fenótipos da hipertensão.^7^ A prevalência de HABNC e HMNC é bastante variável devido à diferença nas condições de tratamento, tipo de medida da pressão arterial fora do consultório e diferença nos critérios de corte das pressões obtidas em casa e no consultório.^18^

Estudo que utilizou a PA de consultório e a média da PA em casa pela manhã e pela noite e adotou os mesmos pontos de corte do presente estudo identificou prevalências mais altas (HMNC 19,0%, HABNC 19,4%, HC 23,0% e HSNC 38,7%). No referido estudo, a maioria dos pacientes com HMNC eram do sexo masculino, mais velhos, fumantes e etilistas, e frequentemente apresentavam um alto IMC, histórico de doenças cardiovasculares e mais complicações do que pacientes com HABNC ou hipertensão controlada.^27^

Pesquisas mundiais de controle da pressão arterial para metas recomendadas por diretrizes nacionais e internacionais revelaram consistentemente que, na prática clínica, a meta convencional de pressão arterial < 140/90 mmHg é alcançada apenas por uma minoria de pacientes.^28^ Uma revisão sistemática mostrou que o controle pressórico varia em torno de 28,4% nos países mais desenvolvidos e apenas 7,7% naqueles com menor grau de desenvolvimento.^29^ No Brasil, a taxa de controle variou de 10,4 a 35,2% em estudo de base populacional.^30^ No atual estudo, a taxa de controle observada foi mais alta que referida pelas outras investigações, chegando a 46,4% de controle (consultório e domicílio).

O Centro de Controle e Prevenção de Doenças observou que aproximadamente 53,5% dos americanos não alcançam a meta de PA.^31^ Embora o monitoramento da pressão arterial no consultório seja o padrão usual de atendimento ou padrão-ouro para diagnóstico e tratamento da hipertensão, o monitoramento da pressão arterial em casa melhora o controle da PA^32^ e a adesão à medicação.^33^ Também já foram demonstradas diversas vezes que a PA domiciliar tem poder preditivo mais forte para mortalidade e morbidade do que a PA medida no consultório.^28 , 34 - 36^ Nos participantes desta pesquisa, o maior controle pressórico foi o domiciliar, sendo observado principalmente no sexo feminino e na faixa etária de 49-60 anos.

Resultados de um estudo sugerem que quase um terço dos pacientes considerados com controle adequado da PA pelos critérios clínicos convencionais não tem a PA controlada quando avaliada fora do consultório. É importante ressaltar que mais de um em cada três pacientes com PA casual limítrofe tem HMNC e, portanto, tem uma PA que não é adequadamente controlada.^37^

Estudo brasileiro observou que as taxas de controle da PA foram de 57,0% pela medida casual e 61,3% pela MRPA (p < 0,001), com prevalência de HABNC e HMNC de 15,4 e 11,1%, respectivamente.^38^ Estudos publicados na última década demonstram que valores de normalidade para MRPA são mais próximos de 130/80 mmHg do que 135/85 mmHg, dando suporte à mudança nos valores de referência de MRPA para 130/80 mmHg.^39^

Em 2020, análise envolvendo 9.868 indivíduos brasileiros não tratados mostrou que valores de PA no consultório, de 140/90 mmHg, corresponderam a valores de 130/82 mmHg na MRPA, enquanto ao analisar 10.069 brasileiros tratados, observou-se que valores de MRPA de 131/82 mmHg são equivalentes a valores da PA no consultório de 140/90 mmHg, e que valores de referência de MRPA mais baixos do que 135/85 mmHg são mais adequados para definir a presença de comportamento anormal da PA.^40^

Assim, a Diretriz Brasileira de HA 2020^11^ recomendou que os valores de anormalidade de MRPA passassem a ser ≥ 130/80 mmHg, em substituição aos valores ≥135/85 mmHg recomendados previamente pela 7^a^ Diretriz Brasileira de HA^1^ e pela 6^a^ Diretriz de Monitorização Ambulatorial da Pressão Arterial e 4ª Diretriz de Monitorização Residencial da Pressão Arterial.^7^ Dados de controle pressórico, com o novo ponto de corte proposto pela DBHA 2020,^11^ ainda não foram relatados na literatura. No referido estudo, percebe-se uma diminuição no controle domiciliar e no número de HABNC, como também um aumento no número de HMNC.

Vários estudos demonstraram que a adição da PA domiciliar ao gerenciamento rotineiro do paciente melhora a adesão ao tratamento, principalmente quando o monitoramento domiciliar da PA é associado à teletransmissão dos valores da PA mensurados pelos pacientes em casa.^41 , 42^ Essa é uma vantagem de suma importância, porque na vida real a baixa adesão ao tratamento é um fenômeno de proporções devastadoras,^43^ que pode ser considerado o principal responsável pelas baixas taxas de controle da PA que caracteriza a população hipertensa^44^ e torna a hipertensão ainda a primeira causa de morte no mundo.^45 , 46^

A obtenção de controle pressórico é crucial para evitar desfechos, como doenças cardiovasculares, insuficiência renal e derrame. Assim, diretrizes recomendam otimizar as dosagens de medicamentos ou adicionar medicamentos anti-hipertensivos até que a pressão arterial alvo seja alcançada.^47 , 48^ A inclusão da PA domiciliar no tratamento de pacientes hipertensos tratados favorece o efeito terapêutico de várias maneiras, ou seja, através de uma melhoria da adesão ao tratamento, evitando o supertratamento e reduzindo a inércia clínica.^46 , 49^

A inércia terapêutica do médico é também uma barreira para que os pacientes atinjam o controle pressórico desejado. Vários motivos podem ser subjacentes aos médicos para que não iniciem ou intensifiquem medicação anti-hipertensiva, incluindo a incerteza em relação à PA do paciente fora do consultório.^50 - 53^ A MRPA incentiva o atendimento centrado no paciente e melhora o controle da pressão arterial e os resultados do paciente.^19^

### Limitações

Este estudo teve algumas limitações. A seleção dos participantes não foi estratificada no sentido de representar a população brasileira de acordo com a população de cada região, podendo assim ter superestimado a região Sudeste. Além disso, a procedência dos pacientes foi apenas de clínicas privadas, podendo não refletir a realidade dos brasileiros que utilizam o Sistema Único de Saúde.

Outra limitação foi o fato de não observar se existia uma correlação entre o controle e o número de medicamentos utilizados, assim como se outros fatores de risco influenciaram ou não o controle pressórico.

## Conclusão

No presente estudo, o controle pressórico nos consultórios em uma amostra de cardiologistas brasileiros foi de 56,3%, levando em consideração a pressão verificada no interior dos consultórios; 61%, quando a PA foi obtida no domicílio; e 46,4% quando o controle foi observado tanto no consultório como no domicílio. A taxa de controle pressórico no domicílio é modificada para 42,4% utilizando-se os novos pontos de corte propostos pela DBHA 2020.
