# Supplementary material for: Registro Nacional do Controle da Hipertensão Arterial Avaliado pela Medida de Consultório e Residencial no Brasil: Registro LHAR
Source: Arq Bras Cardiol. 2023 Jul 21;120(8):e20220863. [Article in Portuguese] doi: 10.36660/abc.20220863 (PMC10464860; doi:10.36660/abc.20220863)
Supplement: Translation

# Translation: National Registry of Hypertension Control Evaluated by Office and Home Measurements: The LHAR National Registry

**Authors:** Roberto Dischinger Miranda, Andréa Araujo Brandão, Weimar Kunz Sebba Barroso, Marco Antonio Mota-Gomes, Eduardo Costa Duarte Barbosa, Lucio Paulo Ribeiro, Claudinelli Alvarenga Aguilar, Fabio Serra Silveira, Cristiano de Melo Rangel Gomes, Abraham Epelman, Annelise Machado Gomes de Paiva, Audes Diógenes Magalhães Feitosa

**Affiliations:** 1 Brasil Serviço de Cardiologia, Disciplina de Geriatria e Gerontologia, Escola Paulista de Medicina, Universidade Federal de São Paulo, São Paulo, SP – Brasil; 2 Brasil Universidade do Estado do Rio de Janeiro, Rio de Janeiro, RJ – Brasil; 3 Brasil Liga de Hipertensão Arterial - Hospital das Clínicas - Universidade Federal de Goiás, Goiânia, GO – Brasil; 4 Brasil Centro Universitário CESMAC, Hospital do Coração, Maceió, AL – Brasil; 5 Brasil Liga de Combate à Hipertensão Arterial, Porto Alegre, RS – Brasil; 6 Brasil Beliva, Recife, PE – Brasil; 7 Brasil Hospital Clínica do Esporte, Goiânia, GO – Brasil; 8 Brasil Centro de Pesquisa Clínica do Coração, Aracaju, SE – Brasil; 9 Brasil Clínica Dicor, Saquarema, RJ – Brasil; 10 Brasil Servier do Brasil, Rio de Janeiro, RJ – Brasil; 11 Brasil Unidade de Hipertensão e Cardiologia Preventiva do PROCAPE, Universidade de Pernambuco, Recife, PE – Brasil

**Keywords:** Hypertension, Blood Pressure, Home Blood Pressure Monitoring, Blood Pressure Control

## Abstract

**Background:**

It is known that around 30% of patients have higher blood pressure (BP) values when examined at the office than at home. Worldwide, only 35% of patients with hypertension undergoing treatment have reached their BP targets.

**Objective:**

To provide epidemiological data on BP control in the offices of a sample of Brazilian cardiologists, considering office and home BP measurement.

**Methods:**

This is a cross-sectional analysis of patients with a hypertension diagnosis and undergoing antihypertensive treatment, with controlled BP or not. BP was assayed in the office by a medical professional and at home using home BP monitoring (HBPM). The association between categorical variables was verified using the chi-square test (p<0.05).

**Results:**

The study included 2540 patients, with a mean age of 59.7 ± 15.2 years. Most patients were women (62%; n=1575). Prevalence rates of 15% (n=382) for uncontrolled white coat hypertension and 10% (n=253) for uncontrolled masked hypertension were observed. The rate of BP control in the office was 56.3% and at home, 61%. Meanwhile, 46.4% of the patients had controlled BP in and outside of the office. Greater control was observed in women and in the 49-61 years age group. Considering the new DBHA 2020 threshold for home BP control, the control rate was 42.4%.

**Conclusion:**

BP control in the offices of a sample of Brazilian cardiologists was 56.3%; this rate was 61% when BP was measured at home and 46.4% when considering both the office and home.

## Introduction

Hypertension is a chronic condition defined by persistently high arterial blood pressure (BP) values which, if not adequately controlled, generate systemic repercussions caused by structural and/or functional injury to target organs. Hypertension is the main modifiable risk factor for cardiovascular and cerebrovascular events. It is considered an important public health problem because of its high and increasing prevalence, low control rates, and high morbidity and mortality.^1 - 4^ The self-reported frequency of a medical diagnosis of hypertension in the adult population of Brazilian capitals and the Federal District is 25.2%, being higher among women (26.2%) than men (24.1%). In both sexes, this frequency increases with age and decreases with schooling levels.^5^

BP measurement is naturally vital to diagnosis. However, despite being a simple procedure, errors can happen during BP measurement and be related to the equipment, the technique, the environment, the patient, or the observer.^6 , 7^ A hypertension diagnosis is given, according to new criteria by the American guidelines, when an individual presents a systolic BP (SBP) ≥ 130 mmHg and/or a diastolic BP (DBP) ≥ 80 mmHg in home, office, and ambulatory measurements.^8^ The most recently published Brazilian guideline, the 2018 European guideline, and the 2020 International Society of Hypertension guideline support the previous criteria, considering an individual to have hypertension if he or she presents SBP ≥ 140 mmHg and/or DBP ≥ 90 mmHg in office measurements. The European and Brazilian guideline, however, brings changes to recommendations of when to consider beginning pharmaceutical treatment according to cardiovascular risk.^1 , 9 , 10 , 11^

It is known that a significant percentage – around 30% – of patients presents higher BP values when examined in the office environment when compared to their homes.^12 - 14^ White coat hypertension (WCH) occurs when persistent BP increases happen in the health care environment and normal values are perceived outside of this environment, leading to an overestimation of the patient’s BP levels and consequent mistakes in BP diagnosis. The opposite of WCH occurs when the patient presents BP levels within the normal range on office measurements, but increased BP outside of the office, which characterizes masked hypertension (MH). In order to differentiate WCH from sustained hypertension (SH) or even to identify the presence of MH, the patient’s BP must be measured outside of the health care environment. Currently used methods include ambulatory blood pressure monitoring (ABPM) and home blood pressure monitoring (HBPM).^13 - 17^

HBPM is the BP measurement performed when the patient is awake, by him or herself or someone with training, with automatic equipment for several days outside the office environment, with predetermined numbers and moments of measurements. HBPM was demonstrated to be the method for diagnosing hypertension that best eliminates the aforementioned effects,^18^ with the additional advantages of presenting good reproducibility, good prognostic capacity, allowing assessment of treatment effects on different periods of the day, relatively low cost, and good acceptance by the patient. A systematic review concluded that both the low sensitivity of office measurements in detecting optimal BP control and the association between HBPM and cardiovascular mortality support the routine use of HBPM in clinical practice.^19^ Studies demonstrated that the use of HBPM when following up patients with hypertension is associated with better adherence to pharmaceutical treatment, with consequent improvement of BP control and reduced cardiovascular outcomes when compared to office BP measurements.^7 , 20^

Less than 15% of the global population with hypertension has reached the recommended BP target, and this rate is as low as 35% among patients with hypertension undergoing treatment.^1^ This fact is highlighted when considering that the BP thresholds recommended by the most recent guidelines^1 , 2 , 8 - 11^ have decreased, which tends to increase the percentage of individuals with uncontrolled hypertension and, consequently, the associated risk of morbidity and mortality by cardiovascular diseases. The first Brazilian registry of hypertension,^21^ using a lower threshold for BP (< 130 × 80 mmHg), found that 24.3% of the general population had controlled BP in the beginning of observation, and 24.7% had controlled BP after 1 year.

Therefore, this study aimed to provide epidemiological data on hypertension control in the medical offices of a sample of Brazilian cardiologists via office BP measurement and HBPM.

## Methods

This is a cross-sectional analysis performed in 231 private health care centers specialized in cardiology and located in 23 Brazilian states and the Federal District, encompassing all regions of Brazil, between June and December 2019. The sample was obtained by convenience sampling and comprised patients who had a medical diagnosis of hypertension, were seen at ambulatory care, were aged ≥ 18 years, and were receiving antihypertensive therapy with a controlled BP or not. In order to avoid selection bias, we asked the physician investigators to invite the second patient seen in each day to participate in the study.

Individuals were initially informed on the objectives and procedures of the research and were then invited to voluntarily participate in the study. After they signed the informed consent form, data collection could begin. This study was approved by the Human Research Ethics Committee of Hospital das Clínicas at Universidade Federal de Goiás with No. 08208619.8.0000.5078.

We collected demographic, clinical, and anthropometric data. The variables date of birth, age, sex, and use of antihypertensive medication were collected through a questionnaire applied during the appointment. Weight and height were obtained using duly calibrated and validated anthropometric scales, and the body mass index (BMI) of adults was classified according to the World Health Organization.^22^

Office BP measurements were performed by physicians according to the recommendations by the VII Brazilian Guidelines of Hypertension (Diretrizes Brasileiras de Hipertensão Arterial [VII DBHA]),^1^ using a suitable cuff for the size of the individual’s arm. Patients with arrhythmia and arm circumferences > 42 cm and < 22 cm did not participate in the study because of limitations of the BP measurement instrument.

HBPM was obtained according to guidance by the IV Guidelines on Home Blood Pressure Monitoring and the European Guidelines on Arterial Hypertension.^7 , 9^ This way, two measurements were made on the first day, still in ambulatory environment (these were not used for analysis of mean home measurement), and home measurements were made in 4 consecutive days, with 3 measurements in the morning and 3 measurements in the night, totaling 24 measurements. The patients were instructed to perform measurements according to a protocol and record them in a BP diary for increasing adherence to the HBPM methodology. The measurements were also recorded and stored in the equipment memory and were then included in the TeleMRPA^®^platform, a telemedicine tool for providing remote reports. Both the office BP measurement and HBPM were obtained using HEM 7320 equipment (Omron Healthcare Co. Ltd., Kyoto Japan).

Participants were considered to have uncontrolled hypertension when SBP ≥ 140 mmHg and/or DBP ≥ 90 mmHg considering office measurements, or when SBP ≥ 135 mmHg and/or DBP ≥ 85 mmHg considering HBPM. Additionally, we analyzed the home BP control rate based on the new thresholds for HBPM, as recommended by the 2020 DBHA.^11^ We used the following terms: masked uncontrolled hypertension (MUCH) for participants who had a controlled BP in the office but increased home or ambulatory measurements; white coat uncontrolled hypertension (WUCH) for those with an increased BP in the office, but controlled home or ambulatory measurements; and SH for those with uncontrolled BP both in the office and ambulatory care. Even though WCH and MH were originally defined for people not receiving treatment for hypertension, they have recently been used to describe discrepancies between BP inside and outside of the medical office for patients being treated for hypertension.^9 , 23^

The database was structured using Microsoft Excel with data from HBPM imported from the recording platform, as well as other data collected by the investigators. Continuous variables are presented as means and standard deviations, whereas categorical variables are presented as relative and absolute frequencies. The association between categorical variables was verified through the chi-square test. We adopted a significance level of p < 0.05. We used SPSS v. 21.0 statistical software (IBM Inc., Chicago, IL, EUA).

## Results

The studied sample included 2540 patients, of which 1.9% (n = 49) were from the North region, 18% (n = 458) were from the Northeast region, 58.2% (n = 1479) were from the Southeast region, 13.5% (n = 342) were from the South region, and 8.3% (n = 211) were from the Central-West region. Of these, 1575 (62%) were female and 965 (38%), male. The mean age was 59.7 ± 15.2 years and the mean BMI, 28.6 ± 5.1 kg/m^2^( Table 1 ).

**Table 1:** – Descriptive characteristics of the sample (n = 2540)

Characteristics	n	%
**Malnutrition**		
Eutrophy	23	0.9
Overweight	590	23.2
Obesity	1070	42.1
Sex	857	33.7
**Female**		
Male	1575	62.0
Masculino	965	38.0
	**Mean**	**SD**
Age (years)	59.7	15.2
BMI (kg/m^2^)	28.6	5.1

Source: this study. BMI: body mass index; SD: standard deviation.

The mean office BP values were 133.3 ± 20.4 mmHg and 82.3 ± 13.2 mmHg; HBPM mean values were 125.9 ± 16.1 mmHg and 78.6 ± 9.3 mmHg for SBP and DBP, respectively. The participants had 14 or more valid measurements at HBPM, where most (94%) participants had a total of 24 valid measurements. The study showed a 15% prevalence (n = 382) of WUCH and a 10% prevalence (n = 253) of MUCH ( Table 2 ).

**Table 2:** – Prevalence of different phenotypes of hypertension (n = 2540)

Phenotypes	n	%
WUCH	382	15
CH	1.168	46
MUCH	253	10
SH	737	29

Source: this study. WUCH: white coat uncontrolled hypertension; CH: controlled hypertension; MUCH: masked uncontrolled hypertension; SH: sustained hypertension.

The prevalence of WUCH among women was 16% (n = 252) and that of MUCH was 8.4% (n = 132); among men, the prevalence rates were 13.5% (n = 130) for WUCH and 12.5% (n = 121) for MUCH. The prevalence of MUCH in men was significantly higher than in women, whereas women presented a higher number of patients with controlled hypertension. Regarding BMI, no statistical difference was observed between hypertension phenotypes; as to the age groups, older individuals (fourth quartile = 70-98 years) presented a higher prevalence of MUCH and the lowest BP control rate ( Table 3 ).

**Table 3:** – Phenotypes vs variables

Phenotype	Sex								p-value*

	Female (n = 1575)				Male (n = 965)				

	n		%		n		%		
**Phenotype**									< 0.01
WUCH	252		16.0		130		13.5		
CH	754		47.9		414		42.9^†^		
MUCH	132		8.4		121		12.5^†^		
SH	437		27.7		300		31.1		
**Phenotype**	**Body Mass Index**								**p-value**
	**Malnutrition (n 23)**		**Eutrophy (n 590)**		**Overweight (n 1070)**		**Obesity (n 857)**		
	**n**	**%**	**n**	**%**	**n**	**%**	**n**	**%**	
**Phenotype**									0.24
WUCH	7	30.4	84	14.2	168	15.7	123	14.4	
CH	8	34.8	269	45.6	511	47.8	380	44.3	
MUCH	1	4.3	56	9.5	108	10.1	88	10.3	
SH	7	30.4	181	30.7	283	26.4	266	31.0	
**Phenotype**	**Age**								**p-value**
	**Q1 (n 650) (18-49 years)**		**Q2 (n 673) (49-61 years)**		**Q3 (n 587) (61-70 years)**		**Q4 (n 630) (70-98 years)**		
	**n**	**%**	**n**	**%**	**n**	**%**	**n**	**%**	
**Phenotype**									< 0.01
WUCH	97	14.9	115	17.1	88	15.0	82	13.0	
CH	319	49.1	325	48.3	284	48.4	240	38.1^‡^	
MUCH	62	9.5	59	8.8	46	7.8	86	13.7^‡^	
SH	172	26.5	174	25.9	169	28.8	222	35.2^‡^	

*p-value for the chi-square test.^†^This prevalence significantly differs from the prevalence in female individuals.^‡^This prevalence significantly differs from the prevalence in female individuals. WUCH: white coat uncontrolled hypertension; CH: controlled hypertension; MUCH: masked uncontrolled hypertension; SH: sustained hypertension.

The rate of BP control among participants of the office research was 56.3% (n = 1431). As to those at home, we observed a control rate of 61% (n = 1550), whereas 46.4% (n = 1178) of the participants in the study had controlled BP both inside and outside of the medical office ( Central Illustration ).

**Central Illustration:** : National registry of hypertension control evaluated by office and home measurements: the LHAR National Registry

Prevalence of overall control.

When considering home BP control stratified by sex ( Figure 1 ) and age ( Figure 2 ), we observed higher control rates among women and in the second quartile (49-61 years age range), respectively.

**Figure 1:** – Prevalence of blood pressure control at home, stratified by sex (p < 0.05).

**Figure 2:** – Prevalence of blood pressure control at home, stratified by age (p < 0.05)*.

The 2020 Brazilian Guidelines of Hypertension^11^ proposed, as normal values for HBPM, 130 mmHg for SBP and 88 mmHg for DBP. Considering this new threshold, the prevalence of patients with WUCH was 7.6% (n = 194); with CH it was 34.9% (n = 886); with MUCH, 21.8% (n = 553); and with SH it was 35.7% (n = 907).

Figure 3 shows the prevalence of BP control in the office and at home, considering the current and previously proposed thresholds.

**Figure 3:** – Prevalence of control with the new thresholds for home blood pressure monitoring.

## Discussion

The diagnosis and treatment of hypertension has been based mainly on office BP measurements. However, BP may differ considerably when measured in the medical office and outside of this environment,^24^ where higher BP values outside of the medical office are associated with increased cardiovascular risk regardless of the office BP.

This study showed that individuals showed increased BP values in the office when compared to measurements made at home. It is known that HBPM values are usually lower than office measurements and are closer to the mean BP recorded during the 24-hour ABPM.^25^

All the studied sample reached the number of valid measurements when performing HBPM. A good quality HBPM fundamentally depends on patient guidance and the use of a measurement diary, eliminating almost 100% of the need for repeating examinations due to insufficient measurements.^13 , 26^

HBPM provides important information on BP levels outside of the office environment in different moments of the day. One of the great advantages of HBPM is the identification and follow-up of hypertension phenotypes.^7^ The prevalence of WUCH and MUCH varies considerably due to differences in treatment conditions, type of BP measurement outside of the office, and thresholds for home and office BP measurements.^18^

A study that used office BP and the mean home BP measurement between the morning and evening, adopting the same thresholds of this study, identified higher prevalence rates (MUCH 19.0%; WUCH 19.4%; CH 23.0%; and SH 38.7%). In this study, most patients with MUCH were male, older, had smoking and alcohol habits, and frequently presented high BMI, history of cardiovascular diseases, and more complications than patients with WUCH or controlled hypertension.^27^

Global research on BP control for thresholds recommended by national and international guidelines consistently revealed that, in clinical practice, the conventional BP target of < 140/90 mmHg is reached only by a minority of patients.^28^ A systematic review showed that BP control varies from around 28.4% in developed countries to only 7.7% in developing countries.^29^ In Brazil, the control rate varied from 10.4% to 35.2% in a population-based study.^30^ In this study, the BP control rate was higher than that reported by other investigations, reaching 46.4% (office and home).

The Centers for Disease Control and Prevention observed that approximately 53.5% of Americans do not reach their BP targets.^31^ Although office BP monitoring is the usual standard of care or gold standard for diagnosing and treating hypertension, HBMP improves BP control^32^ and medication adherence.^33^ Home BP measurements have also recurrently been demonstrated to have stronger predictive power for morbidity and mortality than office BP measurements.^28 , 34 - 36^ Among our study’s participants, the highest BP control rate was seen at home, being observed mainly among women and in the 49-60 years age range.

A study suggests that almost one-third of patients considered to have adequate BP control by conventional clinical criteria do not have controlled BP when evaluated outside of the office. It is important to highlight that more than 1 in 3 patients with borderline casual BP has MUCH, and therefore an inadequately controlled BP.^37^

A Brazilian study observed that BP control rates went from 57.0% by casual measurement to 61.3% by HBPM (p < 0.001), with prevalence rates for WUCH and MUCH of 15.4% and 11.1%, respectively.^38^ Studies published in the last decade demonstrate that normal HBPM values are closer to 130/80 mmHg than to 135/85 mmHg, supporting the change in reference values for HBPM to 130/80 mmHg.^39^

In 2020, an analysis of 9868 untreated Brazilian individuals showed that office BP values of 140/90 mmHg corresponded to HBPM values of 130/82 mmHg, whereas when analyzing 10 069 treated Brazilians, they observed that HBPM values of 131/82 mmHg were equivalent to office BP values of 140/90, and that reference values for HBPM lower than 135/85 mmHg were more suitable for defining the presence of abnormal BP behavior.^40^

Therefore, the 2020 Brazilian Guidelines of Hypertension^11^ recommended that abnormal HBPM values were considered from ≥ 130/80 mmHg instead of ≥ 135/85 mmHg as previously recommended by the VII DBHA,^1^ the VI Guidelines on Ambulatory Blood Pressure Monitoring (Diretrizes de Monitorização Ambulatorial da Pressão Arterial), and the IV Guidelines on Home Blood Pressure Monitoring (Diretriz de Monitorização Residencial da Pressão Arterial).^7^ Data on BP control, with the new threshold proposed by the 2020 DHBA,^11^ have not yet been reported in the literature. In the aforementioned study, a decrease in home BP control and in the number of WUCH cases was observed along with an increase in the number of MUCH cases.

Various studies demonstrated that adding home BP measurements to routine patient management improves treatment adherence, especially when HBPM is associated with the teletransmission of BP values measured by patients at home.^41 , 42^ This is a crucial advantage, since in real life low treatment adherence is a phenomenon of devastating proportions^43^ that can be considered the main responsible for the low BP control rates characterizing the population with hypertension^44^ and making hypertension the main cause of death worldwide.^45 , 46^

Reaching BP control is vital to avoid outcomes such as cardiovascular diseases, kidney failure, and stroke. Therefore, guidelines recommend the optimization of drug doses or the addition of antihypertensive medications until the target BP is reached.^47 , 48^ The inclusion of home BP measurement in the treatment of patients with hypertension favors therapy in various ways, such as with improved treatment adherence, avoiding overtreatment, and reducing clinical inertia.^46 , 49^

Physician inertia is also a barrier to patients reaching the desired BP control. Various reasons could be underlying the physician’s decision to not initiate or intensify antihypertensive medication, including uncertainty of the patient’s BP outside of the office.^50 - 53^ HBPM promotes patient-centered care and improves BP control and patient results.^19^

### Limitations

This study had some limitations. Participant selection was not stratified in the sense of representing the whole Brazilian population according to that of each region, thus it may have overrepresented the Southeast region. Moreover, the patients were originated from private practices, which may not reflect the reality of Unified Health System users.

Another limitation was the fact that we did not observe whether there was a correlation between BP control and the number of drugs being used, or whether other risk factors influenced BP control.

## Conclusion

In this study, the BP control rate in the medical offices of a sample of Brazilian cardiologists was 56.3%, considering the BP verified in office measurements; it was 61% when BP was measured at home, and 46.4% when control was observed both in the office and at home. The home BP control rate changes to 42.4% when using the new thresholds proposed by the 2020 DBHA.
